# Predisposing and protective factors influencing suicide ideation, attempt, and death in patients accessing substance use treatment: a systematic review and meta-analysis protocol

**DOI:** 10.1186/s13643-019-1028-2

**Published:** 2019-05-15

**Authors:** S. Espinet, T. Corrin, D. Baliunas, L. Quilty, L. Zawertailo, S. J. Rizvi, W. deRuiter, S. Bonato, V. De Luca, S. Kennedy, P. Selby

**Affiliations:** 1 Addiction Program, Centre for Addiction and Mental Health, Nicotine Dependence Clinic, (CAMH), 175 College street, Toronto, ON M5T 1P7 Canada; 2 Department of Psychiatry, Campbell Family Mental Health Research Institute, CAMH, University of Toronto, Toronto, Canada; 3 Department of Psychiatry, Arthur Sommer Rotenberg Suicide and Depression Studies Unit, St. Michael’s Hospital, University of Toronto, Toronto, Canada; 4 Department of Library Services, CAMH, Toronto, Canada; 5 Li Ka Shing Knowledge Institute and the Toronto Western Research Institute, University of Toronto, Toronto, Canada

**Keywords:** Substance use, Treatment, Suicidality, Risk factor, Protective factor

## Abstract

**Background:**

The lifetime risk of suicide in patients with substance use disorder is five to ten times the risk in the general population. Critically, up to 19% of patients continue to think about and attempt suicide even after accessing treatment. Therefore, suicidality represents a significant clinical concern in patients struggling with substance use that warrants careful investigation of the factors involved. While most previous research has relied on limited cross-sectional designs, a growing number of prospective studies are improving our understanding of the factors involved. However, a systematic study of these factors has not yet been conducted.

**Methods:**

The primary objective of this review and possible meta-analysis will be to identify key risk and protective factors for suicide ideation, attempt, and death in patients accessing substance use treatment, guided by current models of suicide. Secondary and tertiary objectives will be to obtain pooled effect sizes for the factors identified and to disaggregate factors for suicidality before and after treatment, and for suicidal thought versus action. Following Preferred Reporting Items for Systematic Reviews and Meta-Analyses (PRISMA) guidelines, we will conduct an electronic search of the literature using the databases Embase, Medline, PsycINFO, and Web of Science. Two authors will independently screen studies based on pre-specified inclusion and exclusion criteria, extract relevant data, and assess study quality. Observational and randomized-controlled studies will be included, whereas case-studies and reviews will be excluded. We will extract data on risk and protective factors associated with suicide ideation, attempt (odds or risk ratios), and death (hazard ratio). Given sufficient data (> 5 studies), we will calculate pooled effects using comprehensive meta-analysis.

**Discussion:**

This systematic review will contribute to our knowledge of risk and protective factors for suicidality in patients before and after treatment. Understanding these factors will help define areas of research for further investigation to ultimately inform risk assessment and prevention strategies.

**Systematic review registration:**

PROSPERO (reference number: CRD42018076260).

**Electronic supplementary material:**

The online version of this article (10.1186/s13643-019-1028-2) contains supplementary material, which is available to authorized users.

## Background

Suicide risk represents a salient clinical concern for patients accessing treatment for substance use disorders. Addiction to alcohol and other substances has been established as a major risk factor for suicide across a spectrum of increasingly suicidal behaviors from ideation and attempt to death [1, 2]. Clinical cohort studies indicate that the lifetime risk of suicide death in patients with substance use disorders (SUD) is five to ten times higher than the 3 to 5% risk in the general population [3–5]. While the risk of suicide death among those with alcohol use disorders is ten times that which would be expected in the general population, the risk is 14-fold greater than would be expected with an opiate use disorder and 17-fold greater with mixed drug use [5]. Moreover, the lifetime prevalence of suicide attempt among patients with SUDs ranges from 24 to 78%, and multiple attempts are common [6–10]. Therefore, suicide in the context of substance use treatment (SUT) represents a significant clinical concern that warrants careful investigation of the factors involved.

Clinically treated samples are at risk due to a past history of suicide ideation and attempts, and the risk persists even after treatment. In a cross-national cohort of 34,251 patients with SUDs in the USA, rates of suicide ideation and attempt in the past 30 days prior to treatment were found to be 17% and 3.4%, respectively [9]. Other studies have found rates of suicidal ideation in the year prior to treatment as high as 28.5% for ideation [11] and 10% for attempt [8]. Even after treatment has ensued, a sizeable minority of patients continues to report suicidal ideation (10.4 to 19.9%) and attempts (2.6 to 19%) [6, 8, 11–13]. One possible explanation for these persistently high rates of suicidality may be that treatment for substance addiction that is focused on cessation and relapse prevention may not give adequate attention to the psychosocial risk factors underlying addictive behaviors (cf. [14]).

While a lack of attention to suicide risk factors may explain part of the association between substance addiction and suicidality in treatment, other factors at the individual and socio-contextual levels certainly play a role. Most factors that influence suicide in the general population also influence suicide in people who use substances. These include a previous suicide attempt, pain, social isolation, and hopelessness [15]. However, people who use substances also tend to demonstrate tendencies toward impulsive and aggressive behaviors that influence interpersonal stress and conflict, thereby increasing suicide risk [16, 17]. Substance-related factors such as substance of addiction, addiction severity, recent intoxication and early onset also influence suicide in the context of substance use [18]. Based on a narrative review of the literature on patients with alcohol dependence, Lamis et al. developed a conceptual model of risk factors associated with completed suicide in the context of alcohol use. Unique risk factors included in the model are (1) impulsive and aggressive tendencies, (2) lack of social support, (3) hopelessness, and (4) life strains [16, 17]. The first three of these factors are included as moderators of the link between alcohol use and suicide. Impulsive and aggressive tendencies are modeled as contributing directly to life strains (interpersonal difficulties, negative life events, and alcohol-related problems). Life strains, in turn, are conceptualized as increasing suicide risk both directly, and indirectly, through the promotion of depressive symptoms. While social support is modeled as a mediator of the link between depressive symptoms and suicide risk, according to this model, it is also moderated by hopelessness. Studies that have examined factors for suicidality alongside treatment for other types of substance use have identified additional individual factors, such as victimization and perpetrated violence [19].

Most previous studies aimed at identifying factors associated with high rates of suicidality in patients with SUDs have relied on cross-sectional research designs and retrospective assessment instruments. Therefore, most of what we know about these factors is specific to the pre-treatment context and based on limited study designs. However, a growing number of prospective studies are making important contributions to our understanding of both the risk and protective factors involved. Consistent with previous research, these prospective studies have identified social isolation and depression as key risk factors for future suicide attempt, among other factors such as lifetime history of attempt [12]. However, these studies have advanced research in the field by highlighting additional substance use and treatment-related factors involved. For instance, individuals in SUT for marijuana dependence are less likely to attempt suicide than those in treatment for other substances, whereas those in the same treatment for cocaine or polysubstance use are more likely to attempt suicide [12, 20]. A lack of drug use counseling and emergency treatment are also associated with increased risk of a future attempt [20]. These prospective studies have also begun identifying factors that may protect against suicide in the face of risk. For example, although further study is required to confirm the finding, Ilgen et al. found that mandated treatment acts as a protective factor for suicide attempt, decreasing the risk of an attempt at 1-year follow-up [4].

While the primary literature on suicidality in clinically treated samples is growing, a systematic review is needed to achieve a more comprehensive understanding of the factors involved. Of critical importance is the need to identify protective factors that may reduce the possibility of suicide in the face of immutable risk, consistent with a strengths-based approach emphasizing resilience [21]. Recent intention-to-action models of suicidality also highlight the importance of disaggregating factors for suicidal thought versus action, as factors associated with thought rarely predict the shift to action, although ideation is associated with a subsequent attempt [15]. Thus, the aim of this review will be to fill these gaps by identifying risk and protective factors for suicidality (ideation, attempt, and death) and the strength of these associations, both before and after treatment, taking study design quality into account (cross-sectional versus prospective).

### Objectives

The overall objective of this review is to answer the question: What risk and protective factors influence suicidality (ideation, attempt, death in patients before and after accessing treatment for substance use (SUT))? To this end, the review will address the following three aims:

1. To identify factors for suicidality in patients accessing SUT.
2. To determine the nature (risk or protective) and strength of the association between each of these factors and suicidality.
3. To disaggregate factors for suicidality before and after accessing treatment, as well as factors for suicidal thought (ideation) versus action (attempt and completion).

By providing a better understanding of the individual, social-contextual, and treatment-related factors for suicidality in at-risk individuals seeking SUT, guided by current models of suicide in the general population, it is our intent that this review will contribute to the development of more sensitive risk assessment strategies for clinicians to more accurately predict suicide outcomes. By comparing cross-sectional and prospective studies, this review will also inform theory around risks for suicidality in patients with substance use disorders, by differentiating factors that simply co-occur with suicidal thoughts/actions from those that precede them.

## Methods

This protocol was designed in accordance with established guidelines for systematic reviews and meta-analysis protocols (Preferred Reporting Items for Systematic Reviews and Meta-Analyses Protocols; see Additional file 3) [22] and has been registered in PROSPERO [23].

### Eligibility criteria

The PICO(T) model for clinical questions [24] was used to define the eligibility criteria for this study. This model includes the following components: population, intervention (exposure to risk and protective factors for the purpose of this review), comparison, outcome, and type of study. Specific criteria for including and excluding studies are provided below.

### Population

Studies will be included if they measure suicidality (suicide ideation, attempt, or death) and associated risk or protective factors (as determined by the direction of the pooled associations and confidence intervals) in patients accessing SUT. For the purposes of this review, we have defined SUT in the broadest sense to include any kind of intervention with the intent to modify the addiction regardless of treatment setting or therapeutic elements (including primary and emergency hospital care, community-based peer support such as Alcoholics Anonymous, and methadone maintenance clinics, as examples). No restrictions will be placed on study inclusion based on addiction type, treatment type, participant age, gender, ethnicity, language, nationality, publication language, or status. Studies that are published in a language other than English will be included if the study can be easily translated to English using a web-based translation program; or else, these studies will be excluded. In addition, studies will be excluded that focus on types of addiction other than substance addiction, such as gambling or food addiction, or that target a co-morbid condition (e.g., schizophrenia).

### Exposure

In the context of this review, “exposure” refers to “factors” that increase or decrease the risk of suicidality measured before or after accessing treatment. Studies will be included regardless of the temporal occurrence of the factors with respect to suicidality, as long as the factors occur prior to or concurrent with the suicide outcome(s) of interest. Factors that clearly occur subsequent to the suicidality outcomes of interest will be excluded.

Randomized-controlled studies designed to assess factors for suicidality in SUT, followed by prospective longitudinal studies that measure factors at baseline (treatment entry) and suicide outcome(s) subsequent to treatment entry, will provide the strongest evidence for the causal involvement of these factors. Cross-sectional designs will be considered as providing lower quality evidence, particularly in cases where the factors and suicide outcomes occur concurrently or temporal occurrence is unclear (e.g., measured as “ever occurred”).

Studies designed to compare SUTs that do not consider other individual, socio-contextual, or treatment-related factors for suicidality will be excluded, as will studies that report factors for suicidality in SUT but that were not designed for this purpose.

### Comparator or control

We will include studies that compare patients with the suicide outcomes of interest to patients without these outcomes and studies that compare patients with more severe suicidal behaviors to those with less severe behaviors (e.g., attempters vs. ideators). Studies that compare patients with suicidality in the context of SUT to those from the general population will also be included.

No exclusion criteria will be applied.

### Outcomes

The primary outcome of interest for this review will be suicide ideation: thinking about, considering, or planning to engage in self-directed injurious behavior with an intent to die [15]. Suicide ideation will be examined as the primary outcome because it is the most widely considered outcome in the studies under consideration for this review and because suicide ideation is a key predictor of suicide attempt and death. Therefore, gaining a better understanding of the factors involved in suicidal thinking may limit the potential for these thoughts to lead to action. The secondary outcome of interest will be suicide action, including (1) attempt—nonfatal self-directed, potentially injurious behavior with an intent to die, and (2) completion—death caused by self-directed injurious behavior with an intent to die as a result of the behavior [15]. Assessing suicide outcomes along a continuum of severity is consistent with an intention-to-action framework [15], which will allow for disaggregation of factors associated with suicidal thought versus action.

Studies assessing suicidality using any data source and measurement tool, including self-report and continuous scales, will be included. However, studies examining substance overdose without a clear suicidal intent will be excluded, as will studies that address non-suicidal self-injury or suicide-relevant states such as depression or hopelessness.

### Type of study

Retrospective and prospective observational (cross-sectional, cohort, and case-control), as well as cross-sectional (i.e., analytical) and empirical studies, including randomized-controlled trials (RCTs) will be included in this review. Case studies (case series or case reviews) will be excluded as these study designs do not provide sufficient evidence for the associations of interest to this review. Conference abstracts and unpublished studies will be included if relevant data are provided. Books, letters, reviews, and any articles without relevant, original data will be excluded. Articles to be included may be published at any time, in any geographical location.

### Search strategy

A systematic review of the literature will be conducted using the following electronic bibliographic databases: EMBASE, MEDLINE, Web of Science, and PsycINFO. In collaboration with (SB) who is a librarian and systematic review expert, we developed a comprehensive search strategy for this review using search terms specific to each of the databases, along with free-term texts.

The search strategy is composed of three main search concepts, as follows: (1) suicide (primary outcome of interest); (2) risk and protective factors (exposure); and (3) patients in SUT (population).

For concept 1, we included “suicide” as a medical subject headings (MeSH) term, which includes suicide ideation and attempt, and as a key word. For concept 2, we included the MeSH term “risk factors” and “protective factors.” In addition, we included key words such as “correlate,” “predict,” and “decrease.” Given that this review is focused on patients in SUT, we included concept 3. We divided this concept into two sub-concepts: (1) addiction and (2) treatment. In regards to “addiction,” we included the MeSH headings :“substance-related disorders,” “drug abuse,” “addiction,” and “drug addiction” and key words such as “substance abuse” and “drug dependence.” For the sub-concept “treatment,” we included the MeSH terms “prevention,” and “rehabilitation,” as well as key words such as “treatment,” “intervention,” and “therapy.”

We did not include specific terms for risk or protective factors (e.g., “stress,” “hopelessness,” “social support”), nor did we include specific terms for addiction (e.g., alcohol addiction) or treatment (e.g., relapse prevention), in order to avoid biasing the search toward specific terms and because we assumed that studies including these elements will be selected based on the MeSH terms. We also did not limit the search to specific kinds of studies (e.g., observational or RCTs) to avoid inadvertently screening out potentially relevant studies. The master search strategy created in Embase can be viewed in Table 1.

**Table 1:** Example of complete search strategy using Embase

We developed the master search strategy in Embase on Nov. 5th, 2017 (results: 10,956) using the following search terms:	
1 exp prevention/	
2 exp rehabilitation/	
3 therap$.mp.	
4 intervention$.mp.	
5 treat$.mp.	
6 care.mp.	
7 prevent$.mp.	
8 rehab$.mp.	
9 exp Suicide/	
10 suicid$.mp.	
11 substance-related disorders.mp.	
12 exp Substance-Related Disorders/	
13 exp drug abuse/	
14 exp addiction/	
15 exp drug addiction/	
16 exp “substance use disorder”/	
17 (substance$ adj3 abus$).mp.	
18 (substance$ adj3 addict$).mp.	
19 (substance$ adj3 disorder$).mp.	
20 (substance$ adj3 us$).mp.	
21 (drug$ adj3 disorder$).mp.	
22 (drug$ adj3 us$).mp.	
23 (drug$ adj3 abus$).mp.	
24 (drug$ adj3 dependen$).mp.	
25 (drug$ adj3 addict$).mp.	
26 (drug$ adj3 disorder$).mp.	
27 (addict$ adj3 disorder$).mp.	
28 protective factor$.mp.	
29 exp Protective Factors/	
30 (protect$ adj3 factor$).mp.	
31 protect$.mp.	
32 risk factor.mp.	
33 exp Risk Factors/	
34 (risk$ adj3 factor$).mp.	
35 risk$.mp.	
36 correlat$.mp.	
37 determinant$.mp.	
38 predict$.mp.	
39 associat$.mp.	
40 reduc$.mp.	
41 increas$.mp.	
42 decreas$.mp.	
43 improv$.mp.	
44 enhanc$.mp.	
45 buffer$.mp.	
46 ameliorat$.mp.	
47 1 or 2 or 3 or 4 or 5 or 6 or 7 or 8	
48 9 or 10 (116798)	
49 11 or 12 or 13 or 14 or 15 or 16 or 17 or 18 or 19 or 20 or 21 or 22 or 23 or 24 or 25 or 26 or 27	
50 28 or 29 or 30 or 31 or 32 or 33 or 34 or 35 or 36 or 37 or 38 or 39 or 40 or 41 or 42 or 43 or 44 or 45 or 46	
51 47 and 48 and 49 and 50 (10956)	

NB. We used the following commands specific to the Embase search platform: “Adj3” Search words have to appear at least three words away from each other; “$” Truncation symbol; “exp” Explode the subject heading, to retrieve more specific terms; “/” MeSH heading; “AND,” “OR” Boolean logic to combine search terms

Additional methods for identifying relevant literature will include hand searches of major addiction and suicide journals, and reference lists of included studies. We will also conduct a search of the gray literature (e.g., dissertations, conference abstracts) and will consult with experts in the field who are acting as consultants on this review.

### Data management

We will use DistillerSR to manage references and carry out the review. All articles identified in the searches will first be exported to EndNote X7 and duplicate articles will be identified and deleted. The remaining articles will then be exported to DistillerSR for screening and review.

### Selection procedure

The selection of relevant studies will be carried out independently by two reviewers (SE and TC) at all stages of study selection including screening, full-text review, and quality assessment. Disagreements at any stage of the review will be noted and resolved through consultation with a third, independent reviewer (WdR).

### Article selection

First, a pre-designed relevance screening form (Additional file 1: Appendix 1) will be used to screen titles and abstracts for relevance. Articles that meet the following three inclusion criteria will be reviewed using the pre-designed data extraction and quality assessment form and considered for full-text screening:

1. The study includes suicidality (ideation, attempt, or death) as an outcome, either retrospectively or prospectively.
2. The study examines suicidality as an outcome in patients accessing SUT.
3. Risk or protective factors are assessed in relation to the suicide outcomes of interest.

Second, articles that meet these inclusion criteria will be subjected to a full-text screening and excluded if they meet the following exclusion criteria:

1. The article does not contain original data (e.g., book, letter, review, or case study).
2. The suicide outcome(s) of interest occurred prior to the associated factors.
3. Co-morbid conditions (e.g., gambling and substance addiction) or outcomes (e.g., non-suicidal self-injury and suicide attempt) are assessed and findings are not specific to substance addiction and suicide.

A PRISMA diagram will be created to map the flow of studies through the screening process.

### Data extraction

Articles that meet the inclusion criteria will be independently reviewed by two reviewers (SE and TC). The pre-designed data extraction and quality assessment form (Additional file 2: Appendix 2) will be used by the reviewers to extract relevant information and to assess the quality of the studies. We will extract data in regards to study methodology; treatment type, and setting (e.g., inpatient, community clinic, primary care, group counseling); substance of addiction; statistical methodology; identified risk and protective factors; estimates of association; occurrence and measurement of the suicide outcomes and related factors; and information to assess risk of bias. Care will be taken to record the timing of each factor in relation to each of the associated suicide outcome(s) of interest to ensure that the factors either precede or occur concurrently with the outcome(s). Data from studies that report multiple outcomes (e.g., ideation in the last month and lifetime) and that include multiple follow-up time points will be recorded and included in subgroup analyses. Adjusted measures of association will be recorded and included in the meta-analysis as long as the factors adjusted for are not considered to exist along the causal pathway between substance addiction and suicidality based on the theoretical literature. In cases where measures are adjusted for casual factors, these measures will not be included in the meta-analysis. In addition, when only an unadjusted measure of association is provided, the measure will be recorded and considered for inclusion in the meta-analysis if it is clear that it does not represent an outlier.

### Quality assessment

Quality appraisal of the final studies included in the review will be carried out using the National Institutes of Health, Quality Assessment Tool for Observational Cohort and Cross-Sectional Studies [25]. The tool includes 14 questions designed to capture risk of bias that we have divided into domains similar to the domains included in the Newcastle-Ottawa Scale: (1) selection, (2) exposures, (3) comparability, and (4) outcomes. A total proportion score reported as a percentage will be obtained by dividing the total number of applicable items by the total score obtained across the four domains (see Additional file 2: Appendix 2 for an example of the quality assessment tool). Using these percentage scores, we will assess whether associations differ depending on study quality.

### Data synthesis

A narrative synthesis of the findings related to the risk and protective factors associated with suicidality in the context of SUT will be produced. A summary of each of the studies will also provide data on study location, sample characteristics, suicide outcome rates, substances of use, and treatment types and settings. Particular attention will be paid to identifying the factors involved and classifying them as risk or protective, both (e.g., for instance, a risk factor that on the flipside is also a protective factor), or neither, where applicable. An assessment of the strength of the evidence for causality will also be provided depending on the study design (e.g., prospective studies will provide stronger evidence than cross-sectional). In addition, we will differentiate between factors that were found to be associated with suicidal thoughts only, from those that were associated with suicidal action (attempt and death). We will also differentiate factors found to be associated with suicidality prospectively (after treatment engagement), providing stronger evidence for causality, from those associated with suicidality retrospectively or cross-sectionally (before treatment engagement).

A random-effects meta-analysis will be used to combine results from the studies, or a subset of the studies, if it is appropriate to do so (i.e., studies are homogeneous in regards to design and the factors and outcomes assessed; five or more studies provide data for a particular factor). To assess for heterogeneity (e.g., in study design, location, target sample, treatment, measurement tools, etc.), we will apply the I2 statistic. The strength of the body of evidence (i.e., risk of bias across studies) will be assessed using GRADE [26]. Our data synthesis will also involve the development of a framework to inform preventative strategies, which we will present using a graphical depiction of the factors involved and the strength and evidence of the associations, taking study design and quality into account.

Sub-group analyses (for categorical variables) will also be carried out, if it is appropriate to do so (i.e., ten or more studies provide data on a particular factor), to assess for moderators of the pooled effects. Potential moderators will include study location (e.g., North America, South America, Europe, Asia), publication period (2000 to present vs. prior to 2000), treatment type (licit vs. illicit drugs), study type (prospective or retrospective), and length of follow-up (up to 1 year, 2 to 5 years, 6 to 10 years, 11 or more years).

### Measurement of effect

We will report the strength of the associations between the factors and the suicide outcome(s) of interest as odds ratios (ORs). Both adjusted and unadjusted ORs will be reported when available; however, adjusted ORs will represent the measure of effect. Where these associations are not provided, the group means and variance estimates will be reported. In the case of missing data, we will contact the authors by email using information provided in the publication.

## Discussion

Patients accessing SUT are at heightened risk for suicidality. Clinicians who provide treatment should be equipped with evidence-based assessment and prevention strategies to effectively manage suicide risk. Identifying the risk and protective factors that influence the potential for suicidality in the context of SUT is a critical step in this direction.

Findings from this systematic review may contribute to a preliminary framework for assessing suicide risk in patients accessing SUT based on identified risk and protective factors and the strength of these associations. By identifying prospective factors associated with suicidality and differentiating between factors for suicidal thoughts versus action, this review will potentially contribute theoretically to our understanding of the causal factors involved in influencing increasing levels of suicidality. Findings may inform the management of suicide risk in people with substance addiction with regard to treatment setting, main substance of addiction, gender, age, and treatment phase. The study results also have the potential to inform and expand current theoretical models of substance-related suicide. Furthermore, they may impact clinical approaches to the management of substance use and suicide with important implications for public health policy.

## Additional files

Additional file 1: Appendix 1. Relevance Screening. (DOCX 15 kb)

Additional file 2: Appendix 2. Data Extraction, and Quality Assessment Form. (DOCX 43 kb)

Additional file 3: PRISMA-P 2015 Checklist. (DOCX 26 kb)
