# Supplementary material for: Predisposing and protective factors influencing suicide ideation, attempt, and death in patients accessing substance use treatment: a systematic review and meta-analysis protocol
Source: Syst Rev. 2019 May 15;8:115. doi: 10.1186/s13643-019-1028-2 (PMC6518617; doi:10.1186/s13643-019-1028-2)
Supplement: Supplementary file 1 — Appendix 1. Relevance Screening. (DOCX 15 kb) [file 13643_2019_1028_MOESM1_ESM.docx]

## Appendix 1: Relevance Screening

| Relevance Screening (Study Eligibility) | | |
| --- | --- | --- |
| Does this study examine factors associated with suicidality in individuals receiving addictions treatment, either prospectively (ideally) or retrospectively? | - Yes – relevant research   Check all that apply:   - Risk and/or Protective factors are examined (either deliberately or otherwise) - Suicidality is assessed as an outcome - The study is carried out in the context of addictions treatment - No – not relevant (excluded, submit form) | Addictions Treatment: the application of medicines, psychotherapy, etc, to a patient or to a disease or symptom. This applies to any substance of addiction and can include pharmacology, psychotherapy, maintenance etc.  Suicidality: Death/completion, attempt, planning, ideation, depressive symptoms  Prospective: A prospective cohort study is a longitudinal cohort study that follows over time a group of similar individuals (cohorts) who differ with respect to certain factors under study, to determine how these factors affect rates of a certain outcome.  Risk Factors: Conditions or attributes in individuals, families, communities or the larger society that increase the likelihood of risk or a negative outcome.  Protective Factors: Conditions or attributes in individuals, families, communities or the larger society that help mitigate or eliminate risk. |
