# Supplementary material for: Predisposing and protective factors influencing suicide ideation, attempt, and death in patients accessing substance use treatment: a systematic review and meta-analysis protocol
Source: Syst Rev. 2019 May 15;8:115. doi: 10.1186/s13643-019-1028-2 (PMC6518617; doi:10.1186/s13643-019-1028-2)
Supplement: Supplementary file 2 — Appendix 2. Data Extraction, and Quality Assessment Form. (DOCX 43 kb) [file 13643_2019_1028_MOESM2_ESM.docx]

Appendix 2: Data Extraction, and Quality Assessment Form

| Full-Text Screening (Study Eligibility) | | |
| --- | --- | --- |
| Is this study designed to examine factors associated with suicidality in individuals receiving substance use treatment, either prospectively (ideally) or retrospectively? | - Yes – relevant research - No – not relevant (excluded, submit form) | Addictions Treatment: the application of medicines, psychotherapy, etc, to a patient or to a disease or symptom. This applies to any substance of addiction and can include pharmacology, psychotherapy, maintenance etc.  Suicidality: Death/completion, attempt, planning, ideation, depressive symptoms  Prospective: A prospective cohort study is a longitudinal cohort study that follows over time a group of similar individuals (cohorts) who differ with respect to certain factors under study, to determine how these factors affect rates of a certain outcome.  Risk Factors: Conditions or attributes in individuals, families, communities or the larger society that increase the likelihood of risk or a negative outcome.  Protective Factors: Conditions or attributes in individuals, families, communities or the larger society that help mitigate or eliminate risk. |
| Is a suicide relevant measure assessed (e.g., hopelessness) rather than a measure of ideation, attempt or completion? | - Yes – exclude (excluded, submit form) - No – relevant research |  |
| Is it obvious that the suicidality occurred before the factors? | - Yes – relevant research - No – not relevant (excluded, submit form) |  |
| Indicate the language of publication. Can this publication be translated to English? | Language: [text]   - Yes – relevant research - No – (excluded, submit form) |  |
| What type of Document is this? | Check all that apply:   - Primary research - Thesis - Conference proceeding - Grey literature - Conference abstract with sufficient data - Conference abstract without sufficient data (excluded, submit form) - Book (excluded, submit form) - Review (excluded, submit form) - Case Study (excluded, submit form) - Other, specify: [text] | Primary research: original research/investigation/study carried out by the researcher (includes surveys, interviews, outbreak reports, observations etc.)  Thesis: a long paper/essay or dissertation involving personal research (usually written for a university degree)  Conference proceeding abstract/short paper: an individual or collection of published academic papers  Grey literature: research that is unpublished or published in a non-commercial form (e.g. newspaper or magazine articles)  Case study: in-depth, detailed examination of a subject of study (the case)  Systematic review/meta-analyses: analysis and interpretation of primary research  Literature review: examination of published literature |
| If a co-morbid condition was assessed, is it possible to extract the specific effects of addiction on the suicide outcomes? | - Yes - No (excluded, submit form) - Not applicable |  |

| Data Extraction  General Information | | | |
| --- | --- | --- | --- |
| Question | Options | Definitions/Additional Notes | Source: *pg & ¶/fig/table* |
| What is last name of first authour? | Author: {text} |  |  |
| Indicate the year of publication. | Year: [text] | Year e.g. 1980 |  |
| What is the study location?  *(If not specified, resort to author affiliations)*  Specify the country, state and/or province. | Check all that apply:   - North America - Europe - Australasia - Central - America/South America/Caribbean - Asia - Africa   Specify state and/or province: [text] | North America: includes Canada, USA and Mexico  Europe: includes, Belarus, Latvia, Ukraine, Estonia, Cyprus & west (includes Iceland and Greenland)  Australasia: limited to Australia, New Guinea, New Zealand, New Caledonia, and neighbouring islands, including the Indonesian islands from Lombok and Sulawesi eastward  Central America/South America/ Caribbean: includes Caribbean, and all of south and central America.  Asia: Russia, Turkey, middle eastern countries and east |  |
| What is the study design? | Select one:   - Observational study - Experimental study - Other, specify: [text]   If observational, select one:   - Retrospective - Prospective - Other, specify: [text]   AND  Select one:   - Cohort - Controlled-cohort - Case-control - Cross-sectional - Other, specify: [text]   OR  If experimental select one:   - Randomized control trial - Controlled quasi-experimental study - Pre-/Post-intervention (no control) - Other, specify: [text] | Retrospective: Study looking at suicide as a past event  Prospective: Study looking at suicide as a future event  Cohort: follow a group of exposed and non-exposed individuals to evaluate whether they develop an outcome  Case control: identified cases are compared with controls and their risk factors are evaluated for an association with outcome  Cross-sectional: involve data collected at a defined time to provide data on entire population.  Randomized Control Trial: individuals are allocated at random to a control or intervention group  Controlled quasi-experiment: used to estimate the causal impact of an intervention on its target population without random assignment  Pre-/Post-intervention – examine independent and dependent variable before and after intervention; no control group |  |

| Participants | | | |
| --- | --- | --- | --- |
| Question | Options | Definitions/Additional notes | Source: *pg & ¶/fig/table* |
| Is this study drawn from a larger dataset/study/trial? | Select one:   - Yes, describe: [text] - No - Unclear | Provide any relevant details of larger study including purpose, sample size, design |  |
| Were the inclusion criteria specified, and if so, what are they? | Select one:   - Yes, list the criteria: [text] - No - Unclear |  |  |
| Were the exclusion criteria specified, and if so, what are they? | Select one:   - Yes, list the criteria: [text] - No - Unclear |  |  |
| What is the total sample size for this study? | Provide number: [text] |  |  |
| Is there a control/comparison group and if so, what is the nature and size of this group? | Select one:   - Yes, list the criteria: [text] - No - Unclear - Not Applicable   *Add for each control/comparison* | Copy details from text |  |
| Indicate the number of men and women in the study. | Men: [text]  Women: [text]   - Not stated |  |  |
| What is the average age of the sample in years (provide range or SD) | Average age: [text]  Range or SD: [text] |  |  |
| What was the target population for this study? *(Check all that apply)* | Check all that apply:   - Adolescents, specify: [text] - Adults , specify: [text] - Elderly - Pregnant/parenting women, specify: [text] - Minority groups, specify: [text] - Disadvantaged, specify [text] - Other, specify: [text] - Not stated | Specify any relevant details about the target population (especially details that would render the sample different from the typically population of patients seeking substance use treatment). |  |
| What was the target of substance use treatment for this population? *(check all that apply)* | Check all that apply:   - Tobacco, specify: [text] - Marijuana, specify: [text] - Alcohol, specify: [text] - Amphetamines, specify: [text} - Crack/Cocaine - Opioids, specify: [text] - Polysubstance use, specify: [text] - Other, specify: [text] - Not stated | Tobacco (also Nicotine)  Marijuana (also Cannabis, Hashish)  Alcohol  Amphetamines  Crack (cocaine)  Opioids (heroin, oxycodone, fentanyl, codeine)  Polysubstance use (use of 2 or more drugs)  IV/Injection drug use  Illicit/street drugs |  |

| Treatment | | | |
| --- | --- | --- | --- |
| Question | Options | Definitions/Additional notes | Source: *pg & ¶/fig/table* |
| What was the treatment setting? *(check all that apply)* | Check all that apply:   - Inpatient, specify: [text] - Outpatient, specify: [text] - Residential, specify: [text] - Hospital, specify: [text] - Community Clinic, specify: [text] - Maintenance clinic, specify: [text] - Primary care, specify: [text] - Not stated, specify: [text] - Other, specify: [text] |  |  |
| What were the treatment components? *(Check all that apply)* | Check all that apply:   - Addiction counselling, specify: [text] - Recovery support, specify: [text] - Motivational interviewing, specify: [text] - Social support - Psychoeducation, specify: [text] - Group , specify: [text]interactions/support (e.g., Alcoholics Anonymous), specify: [text] - Parenting support, specify: [text] - Anti-Depressant Medications, specify: [text] - Pharmacological Addiction Treatment, specify: - Other pharmacological treatment, specify: [text] - Other, specify: [text] - Not stated |  |  |
| Who provided the treatment? (Check all that apply) | Check all that apply:   - Doctor, specify: [text] - Nurse, specify: [text] - Social worker, specify: [text] - Psychologist, specify: [text] - Psychiatrist, specify: [text] - Psychotherapist, specify: [text] - Counsellor (NOS) , specify: [text] - other, specify: [text] - Not stated |  |  |
| Describe the treatment (duration [in months], frequency, dosage etc.) | Describe duration/dosage: [text]   - Not stated |  |  |

| Outcomes | | | |
| --- | --- | --- | --- |
| Question | Options | Definitions/Additional notes | Source: *pg & ¶/fig/table* |
| Which suicide outcomes were assessed? | Check all that apply:   - Suicide completion, specify: [text] - Suicide attempt, specify: [text] - Suicide ideation, specify: [text] - Other, specify: [text] | Suicide completion (death due to suicide)  Suicide attempt (e.g., planned, unplanned, impulsive, severe)  Suicide ideation (e.g., plan, thought, intent, discuss) |  |
| How was suicidality assessed? *(specify if varies across outcomes/timepoints)* | - Assessment: [text}   *Add for each outcome* |  |  |
| How was suicidality measured? (e.g., Follow-up 3 occurred six years after end of treatment and the outcome was based on the year prior to assessment) | Check all that apply:   - Pre-treatment, specify: [text] - Baseline, specify: [text] - Post-treatment, specify: [text] - Follow-up 1, specify: [text] - Follow-up 2, specify: [text] - Follow-up 3, specify: [text] - Other, specify: [text] - Not stated   *Add for each outcome* | Pre-treatment – any time before treatment entry  Baseline – treatment entry (no treatment provided)  Post treatment – after baseline but before treatment end  Follow-up 1 – within two years of treatment end  Follow-up 2 – within 5 years of treatment end  Follow-up 3 – within 10 years of treatment end  Other – > 10 years of treatment end |  |
| How many participants experienced the outcome? | Pre-treatment: [n, %]  Baseline: [n, %]  Post-treatment: [n, %]:  Follow-up 1: [n, %]:  Follow-up 2: [n, %]:  Follow-up 3: [n, %]:  *Add for each outcome* |  |  |
| Which factors were examined? | Factor 1: [text]  Factor 2: [text  Factor 3: [text]  *Add for each factor* |  |  |
| How were the factors measured? | - Assessment: [text} - Not stated   *Add for each factor* |  |  |
| For each factor, when was it measured and when did it occur? (e.g., Baseline assessment occurred 3 months after treatment initiation and based on based on the year prior to assessment) | Check all that apply:   - Pre-treatment, specify timing: [text] - Baseline, specify timing: [text] - Post-treatment, specify timing: [text] - Follow-up 1, specify timing: [text] - Follow-up 2, specify timing: [text] - Follow-up 3, specify timing: [text] - Other, specify timing: [text] - Not stated   *Add for each factor* | Pre-treatment – any time before treatment entry  Baseline – treatment entry (no treatment provided)  Post treatment – after baseline but before tx end  Follow-up 1 – within two years of tx end  Follow-up 2 – within 5 years of tx. end  Follow-up 3 – within 10 years of tx. end  Other – > 10 years of tx. end |  |
| Describe the statistical analyses conducted. | Describe: [text] |  |  |
| Were other factors controlled for? | - Yes, specify: [text]   o No  o Unclear |  |  |
| Provide relevant data for each factor in regards to each suicidality outcome | Factor 1, Ideation  Select one:   - Sig., specify: [text] - N.S., specify: [text] - Not stated   Select one:   - Risk, specify: [text] - Prot, specify: [text] - not-applicalble - unclear   Group 1, specify [text]:  *n* [text]  % [text]  Mean [text]  SD [text]  P value [text]  OR select:   - Not applicable   *Add for each group*  Estimate  Select one:   - OR/RR/HR/R^2^, adjusted estimate and CI: [text]; unadjusted estimate and CI: [text] - Other, specify: [text] - Not specified   OR select:   - Not applicable   *Add for each factor and time point* | Sig. = statistically significant (< .05)  N.S. = non-significant (> .05)  Risk factor: Any variable that increases the risk of suicidality by a statistically significant amount (confidence intervals do not overlap with 1.0)  Protective factor: Any variable that decreases the risk of suicidality by a statistically significant amount (confidence intervals do not overlap with 1.0)  *n* = number per group  Mean = average for the group  SD = standard deviation (or other variance estimate)  Estimate = odds ratio, hazard ratio, relative risk or other estimate of effect  CI = confidence interval |  |
| Was there a theoretical basis for assessing these particular set of factors? *(complete for each factor)* | Select one:   - Yes, specify: [text] - No - Unclear |  |  |

| Quality Assessment | | | |
| --- | --- | --- | --- |
| Domain | Risk of bias | Definitions/Additional notes | Source: *pg & ¶/fig/table* |
| Selection |  |  |  |
| Was the research question or objective in this paper clearly stated? | Select one:   - Yes, describe: [text] - No, describe: [text] - CD - NA - NR | Did the authors describe their goal in conducting this research? Is it easy to understand what they were looking to find? This issue is important for any scientific paper of any type. Higher quality scientific research explicitly defines a research question.  CD, cannot determine;  NA, not applicable;  NR, not reported |  |
| Was the study population clearly specified and defined? | Select one:   - Yes, describe: [text] - No, describe: [text] - CD - NA - NR | Did the authors describe the group of people from which the study participants were selected or recruited, using demographics, location, and time period? If you were to conduct this study again, would you know who to recruit, from where, and from what time period? Is the cohort population free of the outcomes of interest at the time they were recruited? |  |
| Was the participation rate of eligible persons at least 50%? | Select one:   - Yes, describe: [text] - No, describe: [text] - CD - NA - NR | If fewer than 50% of eligible persons participated in the study, then there is concern that the study population does not adequately represent the target population. This increases the risk of bias. |  |
| Were all the subjects selected in a way that minimizes selection bias? (randomly assigned or recruited from the same or similar populations including the same time period and communities; inclusion/exclusion criteria pre-specified and uniformly applied to all participants; for case-control recruitment was done independently of intervention or exposure status and sufficient number of cases/controls selected) | Select one:   - Yes, describe: [text] - No, describe: [text] - CD - NA - NR | Were the inclusion and exclusion criteria developed prior to recruitment or selection of the study population? Were the same underlying criteria used for all of the subjects involved? This study recruits groups from different clinic populations, so this example would get a "no."  However, the women nurses described in the question above were selected based on the same inclusion/exclusion criteria, so that example would get a "yes."  RCT: Consider whether researchers describe how the randomization or allocation is generated. A simple statement such as “we randomly allocated” or “using a randomized design” does not suffice. Participants ( researchers ) should be blinded to the randomization sequence)*(R)*  Cohort: consider whether the exposed and non-exposed groups are recruited from the same population *(NR)*  Case-control: consider whether (a) same inclusion and exclusion criteria were applied to cases and controls; (b) whether recruitment was done independently of the intervention or exposure status; and (c) was there a sufficient number of cases/controls selected? *(NR)*  Cross-sectional: consider whether the sample is representative of the population *(NR)*  Circle whether (a) important factors such as age, sex have been evenly distributed across groups; (b) a table lists key demographic information comparing both groups, and there are no obvious dissimilarities between groups that may account for any differences in outcomes, or dissimilarities are taken into account in the analysis |  |
| Exposure |  |  |  |
| For the analyses in this paper, were the exposure(s) of interest measured prior to the outcome(s) being measured? | Select one:   - Yes, describe: [text] - No, describe: [text] - CD - NA - NR | If a cohort study is conducted properly, the answer to this question should be "yes," since the exposure status of members of the cohort was determined at the beginning of the study before the outcomes occurred.  For retrospective cohort studies, the same principal applies. The difference is that, rather than identifying a cohort in the present and following them forward in time, the investigators go back in time (i.e., retrospectively) and select a cohort based on their exposure status in the past and then follow them forward to assess the outcomes that occurred in the exposed and non-exposed cohort members. Because in retrospective cohort studies the exposure and outcomes may have already occurred (it depends on how long they follow the cohort), it is important to make sure that the exposure preceded the outcome.  Sometimes cross-sectional studies are conducted (or cross-sectional analyses of cohort-study data), where the exposures and outcomes are measured during the  same timeframe. As a result, cross-sectional analyses provide weaker evidence than regular cohort studies regarding a potential causal relationship between exposures and outcomes. For cross-sectional analyses, the answer to Question 6 should be "no." |  |
| Was the timeframe sufficient so that one could reasonably expect to see an association between exposure and outcome if it existed? | Select one:   - Yes, describe: [text] - No, describe: [text] - CD - NA - NR | Did the study allow enough time for a sufficient number of outcomes to occur or be observed, or enough time for an exposure to have a biological effect on an  outcome? In the examples given above, if clinical depression has a biological effect on increasing risk for CVD, such an effect may take years. In the other example,  if higher dietary sodium increases BP, a short timeframe may be sufficient to assess its association with BP, but a longer timeframe would be needed to examine its association with heart attacks.  The issue of timeframe is important to enable meaningful analysis of the relationships between exposures and outcomes to be conducted. This often requires at least several years, especially when looking at health outcomes, but it depends on the research question and outcomes being examined.  Cross-sectional analyses allow no time to see an effect, since the exposures and outcomes are assessed at the same time, so those would get a "no" response. |  |
| For exposures that can vary in amount or level, did the study examine different levels of the exposure as related to the outcome (e.g., categories of exposure, or exposure measured as continuous variable)? | Select one:   - Yes, describe: [text] - No, describe: [text] - CD - NA - NR | In any case, studying different levels of exposure (where possible) enables investigators to assess trends or dose-response relationships between exposures and outcomes–e.g., the higher the exposure, the greater the rate of the health outcome. The presence of trends or dose-response relationships lends credibility to the hypothesis of causality between exposure and outcome.  For some exposures, however, this question may not be applicable (e.g., the exposure may be a dichotomous variable like living in a rural setting versus an urban setting, or vaccinated/not vaccinated with a one-time vaccine). If there are only two possible exposures (yes/no), then this question should be given an "NA," and it should not count negatively towards the quality rating. |  |
| Were the exposure measures (independent variables) clearly defined, valid, reliable, and implemented consistently across all study participants? | Select one:   - Yes, describe: [text] - No, describe: [text] - CD - NA - NR | Were the exposure measures defined in detail? Were the tools or methods used to measure exposure accurate and reliable–for example, have they been validated or are they objective? This issue is important as it influences confidence in the reported exposures. When exposures are measured with less accuracy or validity, it is harder to see an association between exposure and outcome even if one exists. Also as important is whether the exposures were assessed in the same manner within groups and between groups; if not, bias may result.  For example, retrospective self-report of dietary salt intake is not as valid and reliable as prospectively using a standardized dietary log plus testing participants' urine for sodium content. Another example is measurement of BP, where there may be quite a difference between usual care, where clinicians measure BP however it is done in their practice setting (which can vary considerably), and use of trained BP assessors using standardized equipment (e.g., the same BP device which has been tested and calibrated) and a standardized protocol (e.g., patient is seated for 5 minutes with feet flat on the floor, BP is taken twice in each arm, and all four measurements are averaged). In each of these cases, the former would get a "no" and the latter a "yes."  Here is a final example that illustrates the point about why it is important to assess exposures consistently across all groups: If people with higher BP (exposed cohort) are seen by their providers more frequently than those without elevated BP (nonexposed group), it also increases the chances of detecting and documenting changes in health outcomes, including CVD-related events. Therefore, it may lead to the conclusion that higher BP leads to more CVD events. This may be true, but it could also be due to the fact that the subjects with higher BP were seen more often; thus, more CVD-related events were detected and documented simply because they had more encounters with the health care system. Thus, it could bias the results and lead to an erroneous conclusion. |  |
| Were the exposure(s) assessed more than once over time? | Select one:   - Yes, describe: [text] - No, describe: [text] - CD - NA - NR | Was the exposure for each person measured more than once during the course of the study period? Multiple measurements with the same result increase our confidence that the exposure status was correctly classified. Also, multiple measurements enable investigators to look at changes in exposure over time, for example, people who ate high dietary sodium throughout the followup period, compared to those who started out high then reduced their intake, compared to those who ate low sodium throughout. Once again, this may not be applicable in all cases. In many older studies, exposure was measured only at baseline. However, multiple exposure measurements do result in a stronger study design. |  |
| Comparability |  |  |  |
| Were key potential confounding variables (including baseline differences) measured and adjusted statistically for their impact on the relationship between exposure(s) and outcome(s)? | Select one:   - Yes, describe: [text] - No, describe: [text] - CD - NA - NR | Logistic regression or other regression methods are often used to account for the influence of variables not of interest. This is a key issue in cohort studies, because statistical analyses need to control for potential confounders, in contrast to an RCT, where the randomization process controls for potential confounders. All key factors that may be associated both with the exposure of interest and the outcome–that are not of interest to the research question–should be controlled for in the analyses. For example, in a study of the relationship between cardiorespiratory fitness and CVD events (heart attacks and strokes), the study should control for age, BP, blood cholesterol, and body weight, because all of these factors are associated both with low fitness and with CVD events. Well-done cohort studies control for multiple potential confounders. |  |
| Outcome |  |  |  |
| Were the outcome measures (dependent variables) clearly defined, valid, reliable, and implemented consistently across all study participants? | Select one:   - Yes, describe: [text] - No, describe: [text] - CD - NA - NR | Were the outcomes defined in detail? Were the tools or methods for measuring outcomes accurate and reliable–for example, have they been validated or are they objective? This issue is important because it influences confidence in the validity of study results. Also important is whether the outcomes were assessed in the same manner within groups and between groups.  Similar to the example in Question 9, results may be biased if one group (e.g., people with high BP) is seen more frequently than another group (people with normal BP) because more frequent encounters with the health care system increases the chances of outcomes being detected and documented. |  |
| Were the outcome assessors blinded to the exposure status of participants? | Select one:   - Yes, describe: [text] - No, describe: [text] - CD - NA - NR | Blinding means that outcome assessors did not know whether the participant was exposed or unexposed. It is also sometimes called "masking." The objective is to look for evidence in the article that the person(s) assessing the outcome(s) for the study (for example, examining medical records to determine the outcomes that occurred in the exposed and comparison groups) is masked to the exposure status of the participant. Sometimes the person measuring the exposure is the same person conducting the outcome assessment. In this case, the outcome assessor would most likely not be blinded to exposure status because they also took measurements of exposures. If so, make a note of that in the comments section.  As you assess this criterion, think about whether it is likely that the person(s) doing the outcome assessment would know (or be able to figure out) the exposure status of the study participants. If the answer is no, then blinding is adequate. |  |
| Was loss to follow-up after baseline 20% or less? | Select one:   - Yes, describe: [text] - No, describe: [text] - CD - NA - NR | Higher overall followup rates are always better than lower followup rates, even though higher rates are expected in shorter studies, whereas lower overall followup rates are often seen in studies of longer duration. Usually, an acceptable overall followup rate is considered 80 percent or more of participants whose exposures were measured at baseline. However, this is just a general guideline. For example, a 6-month cohort study examining the relationship between dietary sodium intake and BP level may have over 90 percent followup, but a 20-year cohort study examining effects of sodium intake on stroke may have only a 65 percent followup rate. |  |
| Was a sample size justification, power description, or variance and effect estimates provided? | Select one:   - Yes, describe: [text] - No, describe: [text] - CD - NA - NR | Did the authors present their reasons for selecting or recruiting the number of people included or analyzed? Do they note or discuss the statistical power of the study? This question is about whether or not the study had enough participants to detect an association if one truly existed. A paragraph in the methods section of the article may explain the sample size needed to detect a hypothesized difference in outcomes. You may also find a discussion of power in the discussion section (such as the study had 85 percent power to detect a 20 percent increase in the rate of an outcome of interest, with a 2-sided alpha of 0.05). Sometimes estimates of variance and/or estimates of effect size are given, instead of sample size calculations. In any of these cases, the answer would be "yes."  However, observational cohort studies often do not report anything about power or sample sizes because the analyses are exploratory in nature. In this case, the answer would be "no." This is not a "fatal flaw." It just may indicate that attention was not paid to whether the study was sufficiently sized to answer a prespecified question–i.e., it may have been an exploratory, hypothesis-generating study. |  |
